# The complete chloroplast genome sequence of *Scurrula parasitica* (Loranthaceae)

**DOI:** 10.1080/23802359.2019.1666684

**Published:** 2019-09-17

**Authors:** Yancai Shi, Ying Zhang, Bingbing Liu

**Affiliations:** a Guangxi Key Laboratory of Plant Conservation and Restoration Ecology in Karst Terrain, Guangxi Institute of Botany, Guangxi Zhuang Autonomous Region and Chinese Academy of Sciences, Guilin, China;; b Institute of Loess Plateau, Shanxi University, Taiyuan, Shanxi, China

**Keywords:** *Scurrula parasitica*, chloroplast genome, phylogenetic analysis

## Abstract

*Scurrula parasitica* Linn. is a hemiparasitic shrub distributed in southern China and Southeast Asian countries. Here, we report and characterize the complete plastid genome sequence of *S. parasitica* in an effort to provide genomic resources useful for the phylogenetic studies for Santalales. The complete chloroplast genome of *S. parasitica* was 122,599 bp in total sequence length, which containing two inverted repeats (IRs) of 23,137 bp separated by a large single-copy (LSC) and a small single copy (SSC) of 70,237 bp and 6,088 bp, respectively. The cpDNA contains 105 genes, comprising 67 protein-coding genes, 30 tRNA genes, 8 rRNA genes. The overall GC content of the plastome is 37.2%. Phylogenetic analysis with 17 species revealed that *S. parasitica* was closely related to the congeneric species *S. notothixoides.*

*Scurrula parasitica* Linn., belonging to family Loranthaceae, is mainly distributed in tropical countries such as Vietnam, Laos, Cambodia, Thailand, Indonesia, and Philippines, as well as in south and southwest China. Recorded hosts for this species include *Citrus aurantium, Cordia dichotoma*, *Euodia lepta*, *Hibiscus tiliaceus*, *Melastoma* sp (Qiu and Gilbert 2003)*. S. parasitica* is wildly used in various traditional Chinese medicine prescriptions. The *Chinese Pharmacopoeia* records it as a medicinal material with wind-dispelling and dampness removal effects; it nourishes the liver and kidneys, strengthens the tendons and muscles, and prevents miscarriage (Liu et al. 2019). Here, we report and characterize the complete plastome of *S. parasitica* based on Illumina paired-end sequencing data, which will contribute to the further studies on its genetic research and resource utilization. The annotated cp genome of *S. parasitica* has been deposited into GenBank with the accession number MN168269.

In this study, *S. parasitica* was sampled from in Jiangxi province of China, located at 114°20′32.04″E, 25°20′25.57″N. A voucher specimen (Y.-C. Shi et al. S205) was deposited in the Guangxi Key Laboratory of Plant Conservation and Restoration Ecology in Karst Terrain, Guangxi Institute of Botany, Guangxi Zhuang Autonomous Region and Chinese Academy of Sciences, Guilin, China. The experiment procedure is as reported in Zhang et al. (2019). Around 2 Gb clean data were used for the cp genome de novo assembly by the program NOVOPlasty (Dierckxsens et al. 2017) and direct-viewing in Geneious R11 (Biomatters Ltd., Auckland, New Zealand). Annotation was performed with the program Plann (Huang and Cronk 2015) and Sequin (http://www.ncbi.nlm.nih.gov/).

The plastome of *S. parasitica* was found to possess a total length 122,599 bp with the typical quadripartite structure of angiosperms, containing two inverted repeats (IRs) of 23,137 bp separated by a large single-copy (LSC) and a small single copy (SSC) of 70,237 bp and 6,088 bp, respectively. The cpDNA contains 105 genes, comprising 67 protein-coding genes, 30 tRNA genes, 8 rRNA genes. Among the annotated genes, 7 of them contain one intron (*rpl*16, *atp*F, *rpo*C1, *trn*L-UAA, *pet*B, *pet*D and *rpl*2), and three genes (*rps*12, *ycf*3, and *clp*P) contain two introns. The overall GC content of the plastome is 37.2%, which is unevenly distributed across the whole chloroplast genome.

To identify the phylogenetic position of *S. parasitica*, phylogenetic analysis was conducted. A neighbor joining (NJ) tree with 1000 bootstrap replicates was inferred using MEGA version 7 (Kumar et al. 2016) from alignments created by the MAFFT (Katoh and Standley 2013) using plastid genomes of 17 species. It showed the position of *S. parasitica* was closely related to the congeneric species *S. notothixoides* (Figure 1). Our findings will provide a foundation for facilitating its genetic research and contributing to its utilization in *Scurrula*.

**Figure 1.**
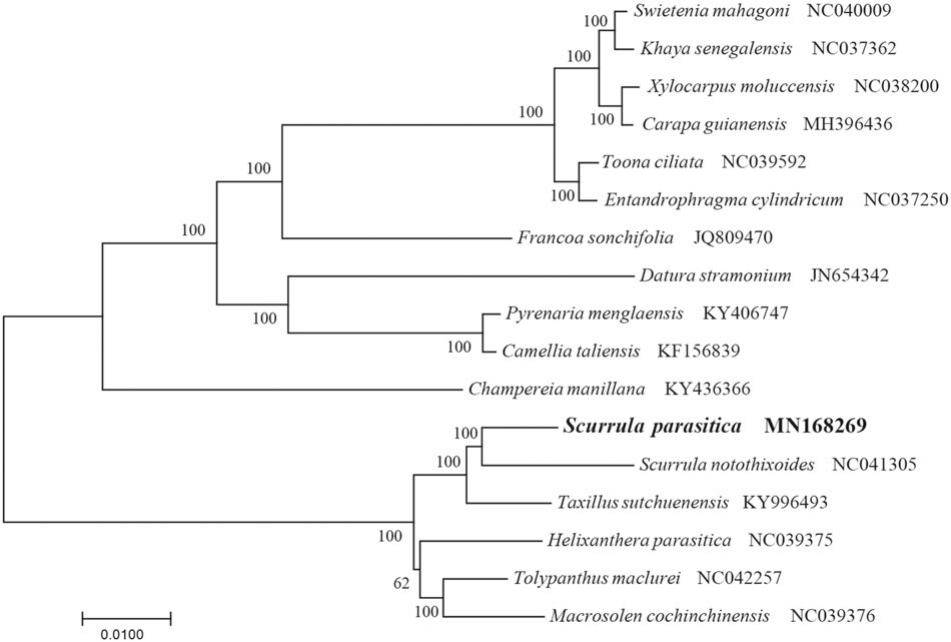
The Neighbour-Joining (NJ) tree based on the 17 chloroplast genomes. The bootstrap value based on 1000 replicates is shown on each node.
